# Synthesis of Honeycomb-Like Co_3_O_4_ Nanosheets with Excellent Supercapacitive Performance by Morphological Controlling Derived from the Alkaline Source Ratio

**DOI:** 10.3390/ma11091560

**Published:** 2018-08-29

**Authors:** Wanli Jia, Jun Li, Zhongjie Lu, Yongfei Juan, Yunqiang Jiang

**Affiliations:** School of Materials Engineering, Shanghai University of Engineering Science, Shanghai 201620, China; wanlian_jia@163.com (W.J.); zhongjielu@outlook.com (Z.L.); 18817307579@163.com (Y.J.); jiangyunqiang2018@163.com (Y.J.)

**Keywords:** hydrothermal method, Co_3_O_4_ nanosheets, mole ratio, electrochemical performance, HMT

## Abstract

Honeycomb-like Co_3_O_4_ nanosheets with high specific surface area were successfully synthesized on porous nickel foam by the facile hydrothermal method followed by an annealing treatment (300 °C), which were used as high-performance supercapacitor electrodes. The effects of the mole ratio of hexamethylenetetramine (HMT) and Co(NO_3_)_2_ (1:1, 2:1, 3:1, 4:1, 5:1 and 6:1) as the reactants on the morphological evolution and electrochemical performance of the electrodes were investigated in detail. X-ray diffractometry (XRD), transmission electron microscopy (TEM), X-ray photoelectron spectroscopy (XPS), and scanning electron microscopy (SEM) were applied to characterize the structure and morphology of the products. The electrochemical performance was measured by cyclic voltammetry (CV) and galvanostatic charge/discharge. The mole ratio of HMT and Co(NO_3_)_2_ produced a significant effect on the morphological evolution of Co_3_O_4_. The morphological evolution of Co_3_O_4_ with the increase in the mole ratio was followed: the nanosheets accompanied with a large number of spherical nanoparticles → the formation of some strip-like particles due to the agglomeration of spherical nanoparticles → the formation of new nanosheets resulting from the growth of strip-like particles → the formation of coarse flower-like particles owing to the connection among the nanosheets → the nanosheets gradually covered with flower-like particles. Accompanied with the change, the specific surface area was increased firstly, and then decreased. A maximum was obtained at a HMT and Co(NO_3_)_2_ mole ratio of 4:1. The evolution in morphology of Co_3_O_4_ was responsible for the change in electrochemical performance of the electrode. The specific capacitance value of the electrode prepared at a HMT and Co(NO_3_)_2_ mole ratio of 4:1 was highest (743.00 F·g^−1^ at 1 A·g^−1^ in the galvanostatic charge/discharge test). The similar result was also observed in the CV test with a scanning rate of 5 mV·s^−1^. Moreover, the electrode also demonstrated an excellent cyclic performance, in which about 97% of the initial specific capacitance remained at 1 A·g^−1^ for 500 cycles in the galvanostatic charge/discharge test. This excellent electrochemical performance was ascribed to high specific surface area of Co_3_O_4_ nanosheets that provide added channels and space for the ions transportation.

## 1. Introduction

The rapid development of the economy and the growth of the global population have depleted conventional energy resources and worsened pollution; thus, the development of new energy has become an urgent need for social progress [1]. Supercapacitors, which are situated between traditional capacitors and batteries, have become the new focus in research on energy sources as a result of their high power density, prolonged life cycle, wide range of operation temperatures, durability under harsh environments, efficient cycling, and low maintenance costs [2]. The electrode material is a decisive affecting factor of the electrochemical performance of supercapacitors. Therefore, investigating the electrode material is a breakthrough point in the exploration of high-performance supercapacitors.

**Figure 1:**
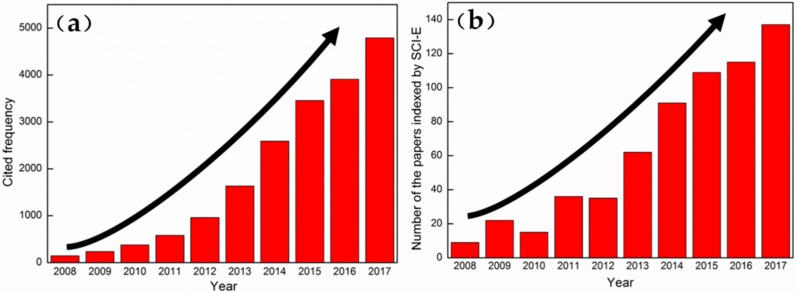
(**a**) The change in the number of papers indexed by SCI-E in 2008–2017; (**b**) The change in the number of cited frequency of those indexed papers from 2008–2017.

Transition-metal oxides (such as RuO [3], MnO_2_ [4], Co_3_O_4_ [5], V_2_O_5_ [6], SnO_2_ [7], TiO_2_ [8], and NiO [9]) have been explored as promising electrode materials. Among them, Co_3_O_4_ is regarded as a favorable candidate for supercapacitors due to its high theoretical capacity (890.00 mAh·g^−1^)/specific capacitance (3560.00 F·g^−1^) [10], comparatively low cost, abundant oxidation states for reversibility, and good environmental affinity [11]. Co_3_O_4_ with different structures (zero-dimensional spherical/octahedral/cubical nanoparticles [12], one-dimensional nanoneedles/nanowires [13,14] with high aspect ratio and nanotubes/nanorods/bundles [15,16] with low aspect ratio, two-dimensional regularly arranged nanosheets [17,18], and regularly-arranged nanobands [19]) has been synthesized by different methods, such as thermal decomposition of solid phase [20], hydrothermal synthesis [21], chemical vapor deposition [22], sol-gel method [23], or electrodeposition [24]. Among these methods, the hydrothermal method is widely used in the preparation of Co_3_O_4_ electrode materials because of its low cost, good crystal shape, high purity, easy operation, high production, and uneasy reunion [25]. From the Science Citation Index Expanded (SCI-E) data, studies on the preparation of Co_3_O_4_ by the hydrothermal method were obtained by the bibliometric method with the “hydrothermal method” and “Co_3_O_4_” as keywords. Figure 1a indicates the change in the number of papers indexed by SCI-E in the last 10 years (2008–2017), whereas Figure 1b shows the change in the number of cited index papers for the same years. Notably, the numbers of papers and cited frequency reveal an increasing tendency with year. The results indicate that the investigation related to the field has become a major task and is gaining increasing attention. However, Co_3_O_4_ metal oxide and its composite materials are usually prepared in powder form, which will be mixed with the appropriate amount of conductive materials (acetylene black) and binders (polytetrafluoroethylene). The slurry will then be placed on different substrates to assemble the electrode [26]. The routine, which is composed of approximately three steps, is comparatively complicated and costly. Moreover, the reliability and conductivity of the electrode are weakened to a certain extent due to the addition of other substances. To solve the above-mentioned shortcomings, Co_3_O_4_ metal oxides have been deposited directly on different substrates in preparing the electrode by the hydrothermal method in recent works. The advantages in the improved method are as follows: (1) Co_3_O_4_ metal oxide is grown directly on nickel foam by the one-step method, which greatly simplifies the preparation process. (2) No additives are introduced into the electrode, thereby considerably reducing the internal/interfacial resistance of the electrode. (3) The inner/outer surfaces, which are found in the nickel foam with a three-dimensional porous structure, will be covered by Co_3_O_4_ metal oxide to improve the electrochemical storage properties, service life, and durability.

Feng et al. [12] investigated the effect of hydrothermal temperature (90–110 °C) on Co_3_O_4_ morphologies. A low temperature (90 °C) did not provide sufficient driving force for Co_3_O_4_ recrystallization. However, Co_3_O_4_ microspheres presented an agglomeration trend as the temperature increased to 105 °C. The optimum hydrothermal temperature was confirmed as 100 °C, at which point the specific capacitances of the prepared Co_3_O_4_ microspheres were 850.00, 780.00, 700.00, and 630.00 F·g^−1^ at current densities of 1, 2, 4, and 8 A·g^−1^, respectively. After 1000 cycles, approximately 90.8% of the specific capacitance was retained at a current density of 2 A·g^−1^. Xia et al. [13] synthesized Co_3_O_4_ nanowires in a system comprising 30 mmol of Co(NO_3_)_2_ and 3 mmol of sodium nitrate at 120 °C for 12 h, which was then annealed in flowing argon at 250 °C for 1 h. The product exhibited specific capacitances of 599.00 F·g^−1^ at 2 A·g^−1^ and 439.00 F·g^−1^ at 40 A·g^−1^. Chang et al. [14] also prepared Co_3_O_4_ nanowires at a cobalt glucose/Co(NO_3_)_2_·6H_2_O mole ratio of 1:2 by the hydrothermal method, followed by an annealing treatment at 300 °C. The sample exhibited a specific capacitance of 471.80 F·g^−1^ at 0.5 A·g^−1^ and cycling stability with 94.8% capacitance retention after 1000 cycles at 2 A·g^−1^. Venkatachalam et al. [15] synthesized Co_3_O_4_ nanorods via a hydrothermal reaction (120 °C for 12 h) between cobalt chloride (CoCl_2_·6H_2_O) and urea [CO(NH_2_)_2_] and then annealed the nanorods at 300 °C for 5 h. The modified Co_3_O_4_ electrode exhibited a specific capacitance of 655.00 F·g^−1^ at a current density of 0.5 A·g^−1^. Mesoporous Co_3_O_4_ bundles were synthesized by the hydrothermal method (180 °C for 24 h) followed by an annealing treatment (350 °C for 3 h) [16]. The specific capacitance of the Co_3_O_4_ bundles was 357.00 F·g^−1^ at 2 A·g^−1^ after 500 cycles. Moreover, the mesoporous Co_3_O_4_ was found to maintain 85.5% of specific capacitance with an increase in the current density from 1 A·g^−1^ to 20 A·g^−1^. Co_3_O_4_ nanoflakes were successfully synthesized by the hydrothermal method (90 °C for 24 h) and then annealed (300 °C for 4 h) [17]. The charge−discharge measurement showed that the specific capacitance of these electrodes was 263.00 F·g^−1^ within a potential range of −0.40–0.55 V, and the Co_3_O_4_ flakes retained 89.4% of specific capacitance at 3 A·g^−1^ after 1000 consecutive cycles. Duan et al. [18] prepared a two-layer Co_3_O_4_ film on a self-assembled monolayer polystyrene sphere template by the hydrothermal method (100 °C for 2 h), followed by annealing (250 °C for 1.5 h). The average specific capacitance, which was calculated from the cyclic voltammetry (CV) curve, was approximately 360.00 F·g^−1^ at a scanning rate of 10 mV·s^−1^, and a value of 454.00 F·g^−1^ was retained at 2 A·g^−1^ after 2500 cycles.

Present studies mainly focus on the improvement in specific capacitance of Co_3_O_4_ with different morphologies by regulating the affecting factors, such as hydrothermal temperature and reactant components and their ratios. On the basis of this notion, a correlation between morphologies and affecting factors was established to obtain Co_3_O_4_ with a high specific capacitance. Table 1 summarizes the specific capacitance of Co_3_O_4_ as reported in related references. The table shows that the value of Co_3_O_4_ ranges from 100.00 F·g^−1^ to 850.00 F·g^−1^ and is less than its theoretical value (approximately 3560.00 F·g^−1^). Therefore, there is a large space for improving the specific capacitance of Co_3_O_4_.

To date, hexamethylenetetramine (HMT) and Co(NO_3_)_2_ are frequently used reactants for the hydrothermal synthesis of Co_3_O_4_. However, investigations on the effect of their ratio on the morphological evolution of Co_3_O_4_ are few. Thus, the precise optimum ratios of these reactants remain unidentified. In our work, HMT and Co(NO_3_)_2_ with different mole ratios (1:1, 2:1, 3:1, 4:1, 5:1 and 6:1) were used for the hydrothermal synthesis of Co_3_O_4_ with high specific surface area. The morphological evolution of Co_3_O_4_ with the changes in the HMT/Co(NO_3_)_2_ mole ratio was investigated in detail. Moreover, the nucleation and growth mechanisms of Co_3_O_4_ were revealed. On the basis of the obtained results combined with the measurement results of specific capacitance, the optimum mole ratio was finally determined.

## 2. Materials and Methods

### 2.1. Synthesis of Co_3_O_4_ Nanosheets on Nickel Foam

**Figure 2:**
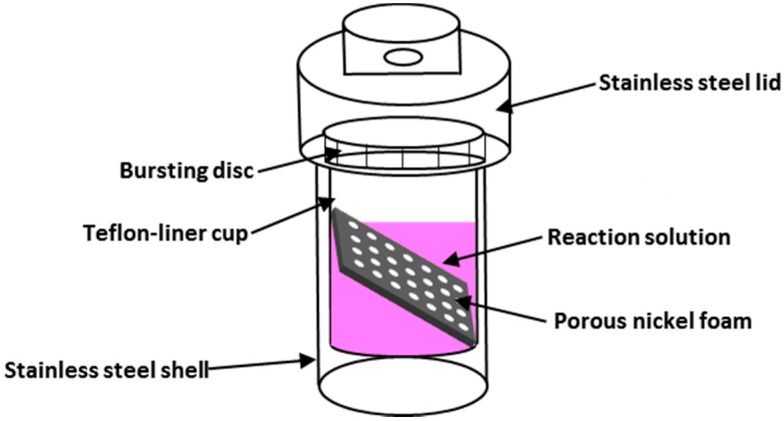
Schematic illustration of the Teflon-liner stainless steel autoclave.

All the reagents in the experiments were of analytical grade and were used without further purification. Nickel foam (2.5 cm × 3.5 cm) was selected as the electrode substrate (Shanxi Powder Source Battery Materials Co., Ltd., Taiyuan, China) and cleaned by a 3 M of HCl solution for 30 min to remove the NiO layer surface and then washed with deionized water and ethanol several times in an ultrasonic bath. Thereafter, the samples were dried at 60 °C and weighted using an electronic balance (0.01 mg, Sartorius SQP, Beijing, China). Next, 2 mmol of Co(NO_3_)_2_·6H_2_O was added into a mixed solvent consisting of deionized water (20 mL) and absolute ethanol (10 mL) under magnetic stirring, and different moles of HMT (2, 4, 6, 8, 10, and 12 mmol) were added into the solution. Solutions with six HMT/Co(NO_3_)_2_ mole ratios were prepared (1:1, 2:1, 3:1, 4:1, 5:1, and 6:1). The pink solutions were transferred into six 50 mL Teflon-lined stainless-steel autoclaves (Figure 2), and six pieces of nickel foam were immersed into the solutions. The autoclaves were sealed and maintained at 90 °C for 12 h and then cooled to room temperature. The obtained samples were rinsed several times with deionized water and dried at 60 °C for 2 h. The samples were annealed to obtain Co_3_O_4_ nanosheets for 2 h at 300 °C in air with a heating rate of 2 °C·min^−1^ and were weighted again. The difference between the two weights was the amount of the active substances. Six samples, which were prepared at different mole ratios, were labeled as Samples A (1:1), B (2:1), C (3:1), D (4:1), E (5:1), and F (6:1).

### 2.2. Material Characterization

The evolution in phase constituent of the samples before and after annealing was identified with an X-ray diffractometer (XRD, D2-PHASER Bruker, Karlsruhe, Germany) using Cu Kα radiation (γ = 0.1540560 nm). The elemental compositions and chemical states of the Co_3_O_4_ precursors and their sintered products were further determined with an X-ray photoelectron spectrometer (XPS, ESCALAB 250Xi, Thermo Fisher Scientific, Waltham, MA, USA). Morphologies of the annealed samples were observed under a field-emission scanning electron microscope (FESEM, S-4800, Hitachi, Tokyo, Japan). Information on the crystal structure was identified under a transmission electron microscope (TEM, JEM-2100F, JEOL, Tokyo, Japan). Chemical compositions were confirmed with an energy dispersive spectrometer (EDS, IET 200, Oxford Instruments, Oxford, UK).

### 2.3. Electrochemical Performance

The electrochemical performance of the Co_3_O_4_ electrodes was measured by CV and galvanostatic charge-discharge tests on a CHI 6082D (CH Instrument Inc., Shanghai, China). A 2 M of KOH aqueous solution was used as the supporting electrolyte. A three-electrode system was applied. The nickel foam piece covered with Co_3_O_4_ was used as the working electrode. The graphite sheet and saturated calomel electrode (SCE) were selected as the counter and reference electrodes, respectively. CV scans were recorded from −0.2 V to 0.6 V (vs. SCE) at scanning rates from 5 mV·s^−1^ to 100 mV·s^−1^, and the cycling stability of the Co_3_O_4_ electrodes was evaluated by CV for 500 cycles at 50 mV·s^−1^. Galvanostatic charge-discharge tests were performed at different constant current densities (1, 2, 4, 6, 8, and 10 A·g^−1^).

## 3. Results and Discussion

### 3.1. Formation Mechanism of Co_3_O_4_

When the reaction system including Co(NO_3_)_2_·6H_2_O and HMT is heated, HMT will be decomposed into NH_3_ and formaldehyde by Equation (1) [18]. A portion of NH_3_ will further react with H_2_O to increase the concentration of OH^−^ in the solution by Equation (2):

(1) $$C_{6}H_{12}N_{4}+6H_{2}O\to 6\mathrm{HCHO}+4{\mathrm{NH}}_{3}$$

(2) $${\mathrm{NH}}_{3}+H_{2}O↔{\mathrm{NH}}_{4}^{+}+{\mathrm{OH}}^{-}$$

In an alkaline solution rich in Co ions, nickel foam as the substrate will play an important role in the synthesis of Co(OH)_2_ due to its surface covered with some intermediate products. Some investigations proved that the nanowire-like Ni-Co-based carbonate hydroxide is formed preferentially on the surface of nickel foam in the hydrothermal process [34,35,36]. A large number of the intermediate products adhering to the nickel foam surface possess considerably high surface energy, owing to their very high specific surface area. The heterogeneous nucleation centers significantly promote the deposition of Co(OH)_2_. The intermediate products synthesized in the hydrothermal process will be transformed into spinel NiCo_2_O_4_ nanowires in subsequent annealing. Du et al. [34] prepared the precursor on nickel foam in an alkaline solution (1 mmol Ni(NO_3_)_2_·6H_2_O, 2 mmol CoCl_2_·6H_2_O and 15 mmol HMT dissolved into 70 mL water) by the hydrothermal method. Then, the precursor was annealed to obtain NiCo_2_O_4_ nanowires. Cai et al. [35] synthesized NiCo_2_O_4_ nanosheets on nickel foam in a solution (2 mmol Co(NO_3_)_2_·6H_2_O, 1 mmol NiCl_2_·6H_2_O, 6 mmol NH_4_F and 10 mmol HMT and 30 mL deionized water) by the same method. Wang et al. [36] prepared the nanowire-like Ni-Co-based carbonate hydroxides on nickel foam by hydrothermal precipitation in an alkaline solution containing Co(NO_3_)_2_·6H_2_O, Ni(NO_3_)_2_·6H_2_O, NH_4_F, and urea. The products were further converted into NiCo_2_O_4_ nanowires growing directly on the substrate by the annealing treatment. Besides the high specific area of the intermediate substance, the difference in isoelectric points between it and Co(OH)_2_ is also responsible for the synthesis of Co(OH)_2_ since it directly affects the electrostatic interaction between them [37]. When the pH value in the solution is between the two isoelectric points of two substances, the nucleation of Co(OH)_2_ on the intermediate substance will be accelerated due to their electrostatic attraction occurring between opposite electrical charges. Although no precise isoelectrc points of the two substances are obtained, there is no doubt that the isoelectric point is a non-negligible factor affecting the synthesis of Co(OH)_2_.

**Figure 3:**
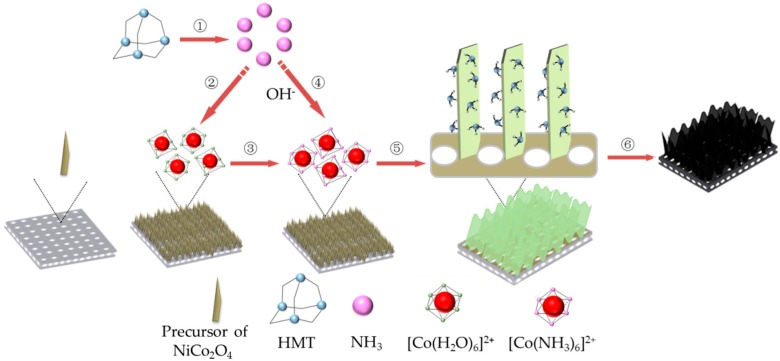
Schematic illustration of the synthesis process for Co_3_O_4_ nanosheets.

The formation mechanism of Co_3_O_4_ can be described intuitively by a schematic illustration (Figure 3). With Equation (1) in progress, the concentration of NH_3_ will be constantly increased in the sealed system. According to coordination theory [38], most metal ions form metal complexes with an octahedral structure with the presence of a large number of ligands, such as H_2_O and NH_3_, in the solution. According to the electronic theory of acid and alkali [39], the alkalinity of NH_3_ is stronger than H_2_O. The ligand H_2_O around Co (II) in [Co(H_2_O)_6_]^2+^ will be inevitably replaced by NH_3_ to form a metal complex with an octahedral structure. This notion is due to the fact that [Co(NH_3_)_6_]^2+^ has a higher complex stability coefficient than [Co(H_2_O)_6_]^2+^ [40], in which Co^2+^ and NH_3_ are located at the center and six vertex positions of the octahedron. The process can be described as follows:

(3) $${\left[\mathrm{Co}{\left(H_{2}O\right)}_{6}\right]}^{2+}+6{\mathrm{NH}}_{3}\to {\left[\mathrm{Co}{\left({\mathrm{NH}}_{3}\right)}_{6}\right]}^{2+}+6H_{2}O$$

Xia et al. [13] prepared self-supported hollow Co_3_O_4_ nanowires by a facile hydrothermal synthesis method. [Co(H_2_O)_6_]^2+^ was confirmed to first react with NH_3_ to generate [Co(NH_3_)_6_]^2+^ in the presence of NH_3_, which affected the subsequent reaction directly. Feng et al. [41] also proposed that [Co(H_2_O)_6_]^2+^ underwent a reconstruction process by the introduction of NH_3_ under hydrothermal conditions, so that Co_3_O_4_ was prepared at relatively low temperatures. This indicates that the generation of NH_3_ resulting from Equation (1) is a very influential factor in the synthesis of Co_3_O_4_. [Co(NH_3_)_6_]^2+^ gradually becoming the dominant substance in alkaline solution with the reconstitution that constantly occurs under hydrothermal conditions, which will greatly promote the two-dimensional instantaneous nucleation [38]. Accompanied with this, the concentration of OH^−^ in the solution is also increased in the solution due to the excessive concentration of NH_3_ (see Equation (2)), which is also conducive to the formation of the membrane structure [42,43]. As a result, the complex [Co(NH_3_)_6_]^2+^ will react with OH^−^ to generate Co(OH)_2_ with high stability via the following reaction [18,44]. During the subsequent annealing, Co(OH)_2_ is decomposed into Co_3_O_4_ nanosheets.

(4) $${\left[\mathrm{Co}{\left({\mathrm{NH}}_{3}\right)}_{6}\right]}^{2+}+2{\mathrm{OH}}^{-}\to \mathrm{Co}{\left(\mathrm{OH}\right)}_{2}↓+6{\mathrm{NH}}_{3}$$

### 3.2. Chemical Compositions and Phase Constituents of the Products

**Figure 4:**
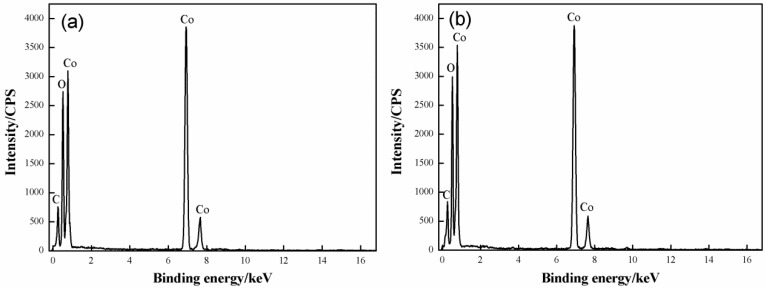
EDS results of the samples: (**a**) sample D; (**b**) sample F.

EDS was used to identify the chemical compositions of Samples D and F. Figure 4 provides the results. The chemical compositions of Samples D and F are very similar; specifically, the two samples are rich in Co (40 at.%; at.% is percentage of the atom), O (60 at.%), and C (7.3 at.%). In conclusion, the synthesized product is composed of oxides that contain Co.

**Figure 5:**
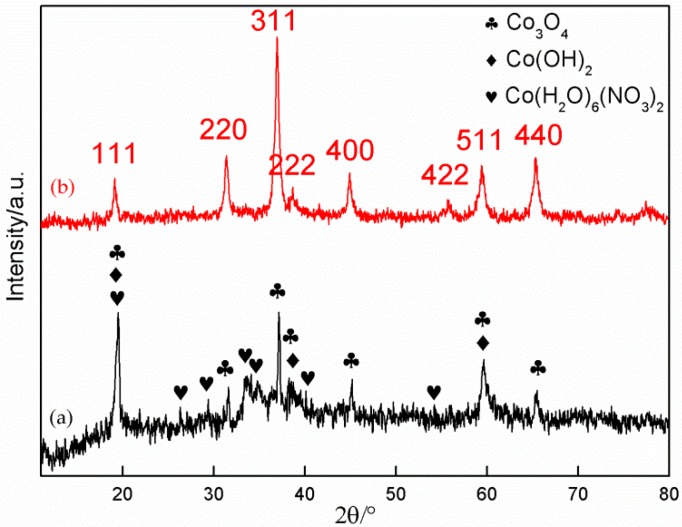
XRD patterns of sample D: (**a**) before annealing; (**b**) after annealing.

Figure 5 indicates the XRD patterns of the synthesized product with a 4:1 mole ratio of HMT/Co(NO_3_)_2_ before and after annealing. A large number of diffraction peaks with different intensities can be observed in the XRD pattern of the initial product. The indexed result shows that the product is mainly composed of Co(OH)_2_ (JCPDS01-074-1057) and Co_3_O_4_ (JCPDS01-076-1802). Moreover, traces of the reactants (Co(NO_3_)_2_·6H_2_O, JCPDS01-071-0726) are found in the product. The synthesis of Co(OH)_2_ has been previously discussed. Given the remaining oxygen in the Teflon-lined stainless-steel autoclaves, a portion of Co(OH)_2_ products were further oxidized to Co_3_O_4_ via the following reaction at high temperature [33].

(5) $$6\mathrm{Co}{\left(\mathrm{OH}\right)}_{2}+O_{2}\to 2{\mathrm{Co}}_{3}O_{4}+6H_{2}O$$

After the product was annealed for 2 h at 300 °C, the XRD pattern presents a considerable change. The pattern becomes smoother than before, and the diffraction peaks related to Co(NO_3_)_2_·6H_2_O nearly disappear. Therefore, Co(NO_3_)_2_·6H_2_O has been completely decomposed into Co_3_O_4_ and gases (NO_2_ and O_2_) via the following reaction:

(6) $$3\mathrm{Co}{\left({\mathrm{NO}}_{3}\right)}_{2}\to {\mathrm{Co}}_{3}O_{4}+6{\mathrm{NO}}_{2}+O_{2}$$

From the changes in intensity of three strong peaks related to Co_3_O_4_ and Co(OH)_2_, we can conclude that the content of Co(OH)_2_ is considerably reduced after annealing due to its conversion into Co_3_O_4_ by Equation (5) at high temperature.

**Figure 6:**
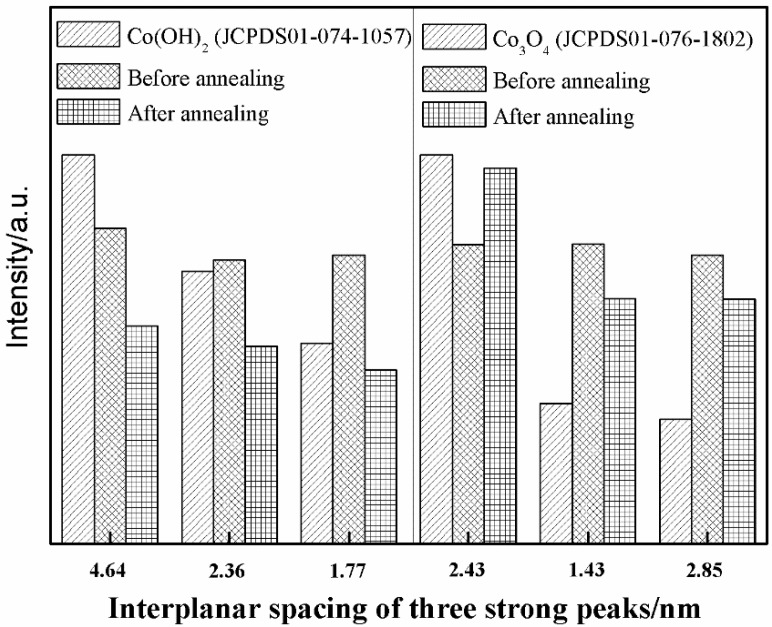
Change in intensity of three strong peaks related to Co(OH)_2_ and Co_3_O_4_ before and after annealing.

Identifying whether Co(OH)_2_ is completely oxidized to Co_3_O_4_ is extremely difficult because the main diffraction peaks related to Co(OH)_2_ nearly coincide with those related to Co_3_O_4_. However, the changes in intensity of the diffraction peaks related to Co(OH)_2_ and Co_3_O_4_ prove that the remainder of Co(OH)_2_ has been greatly reduced (even completely removed) in the product after annealing. Figure 6 shows the changes in three strong peaks of Co(OH)_2_ and Co_3_O_4_. The intensities of the three strong peaks of Co(OH)_2_ are obviously decreased after annealing compared with those in the initial product. Therefore, the Co(OH)_2_ content has been remarkably reduced. Given that the three strong peaks are also related to Co_3_O_4_, they have comparatively high intensities after annealing. On the contrary, the intensity of the strongest diffraction peak of Co_3_O_4_ is enhanced from 76.94% to 98.68% after annealing, which imply that the majority of Co(OH)_2_ has reacted with oxygen.

**Figure 7:**
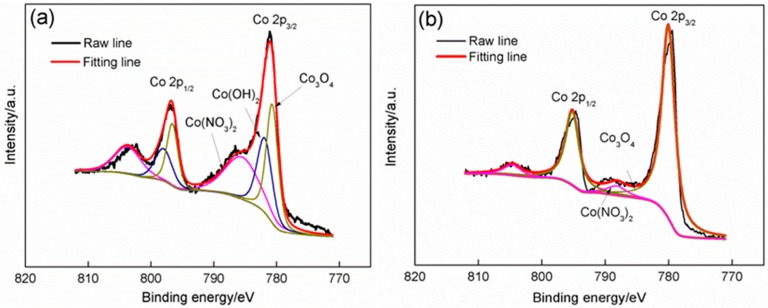
High resolution XPS spectra of Co 2p in sample D: (**a**) before annealing; (**b**) after annealing.

The chemical compositions of Sample D were further analyzed by XPS (Figure 7a,b). The spectroscopic spin orbit of Co 2p can be divided into 2p_1/2_ and 2p_3/2_, which contain the same chemical information. Obvious differences between the two XPS spectra before and after annealing are noted. Four peaks can be clearly observed in the XPS spectra before annealing, which are located at 803.01, 796.87, 786.60, and 781.08 eV. According to the XPS binding energy manual, these peaks can be fitted into three pairs (Figure 7a). Two peaks situated at 803.01 and 786.60 eV agree well with those of Co in Co(NO_3_)_2_ with standard binding energies of 803.05 and 786.85 eV. The two other peaks with binding energies of 796.87 and 781.08 eV can be confirmed as combinations of two pairs related to Co(OH)_2_ and Co_3_O_4_ (standard binding energies for Co in Co(OH)_2_: Co 2p_1/2_ 798.00 eV, Co 2p_3/2_ 781.00 eV; for Co in Co_3_O_4_: Co 2p_1/2_ 795.90 eV, Co 2p_3/2_ 780.10 eV). We conclude that the product is mainly composed of Co(OH)_2_ and Co_3_O_4_ along with small amounts of Co(NO_3_)_2_. After the product is annealed, a pair of peaks that are related to Co(NO_3_)_2_ nearly disappear due to the considerable reduction in their intensities. Moreover, the two other peaks shift toward the right, which results in their binding energies decreased from 796.87 eV to 794.80 eV for the first peak, from 781.08 eV to 779.61 eV for the second peak. This change makes the two peaks only match with those related to Co in Co_3_O_4_. It further demonstrates that the intermediate product (Co(OH)_2_) and residual reactant (Co(NO_3_)_2_) are nearly completely converted into Co_3_O_4_ after annealing.

**Figure 8:**
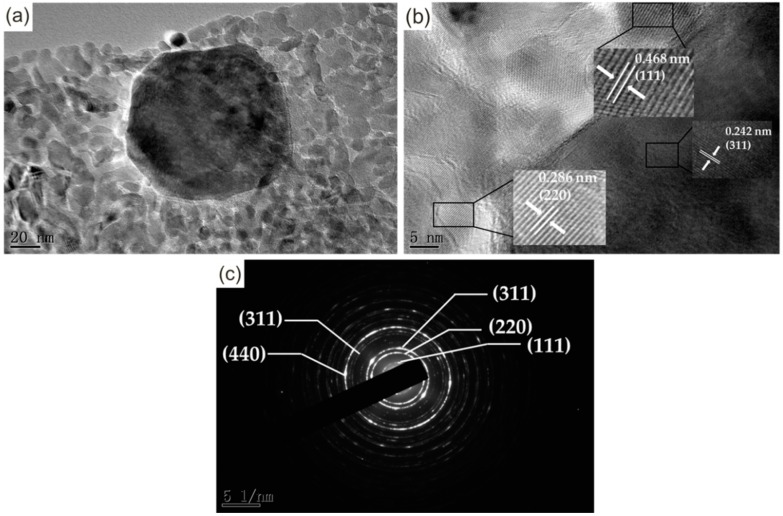
(**a**) Transmission electron microscope (TEM) image; (**b**) Selected area electron diffraction; and (**c**) high-resolution transmission electron microscope (HRTEM) image of the annealed product prepared at a HMT/Co(NO_3_)_2_ mole ratio of 4:1.

TEM was applied to acquire precise structure information of the annealed product prepared in HMT/Co(NO_3_)_2_ at a mole ratio of 4:1. Figure 8a illustrates the morphology of the product. A large number of equiaxed or near-equiaxed particles with smooth edges cluster together, among which numerous interfaces can be clearly observed. A large particle with a size of approximately 80 nm is found, and the sizes of the rest are located at a range of approximately 5–30 nm. Notably, the contrast in different zones of the particles is nearly similar. From the formation mechanism of the mass thickness contrast, we can conclude that nanosheets with similar thickness are successfully synthesized. The lattice fringes are shown in a HRTEM image (Figure 8b). The plane distances in different zones are calculated to be 0.242, 0.286, and 0.468 nm for the nanosheets, and they fully match with the (311), (220), and (111) planes of Co_3_O_4_. No lattice fringes related to Co(OH) are observed, which further confirms that Co(OH)_2_ is completely oxidized to Co_3_O_4_. Figure 8c presents the selected area electron diffraction pattern of the nanosheets. Five concentric diffraction rings from the center can be observed. It has been demarcated with the Co_3_O_4_ interplanar crystals (111), (220), (311), (422), and (440), which are completely consistent with the XRD result.

### 3.3. Morphological Characterization of Co_3_O_4_

**Figure 9:**
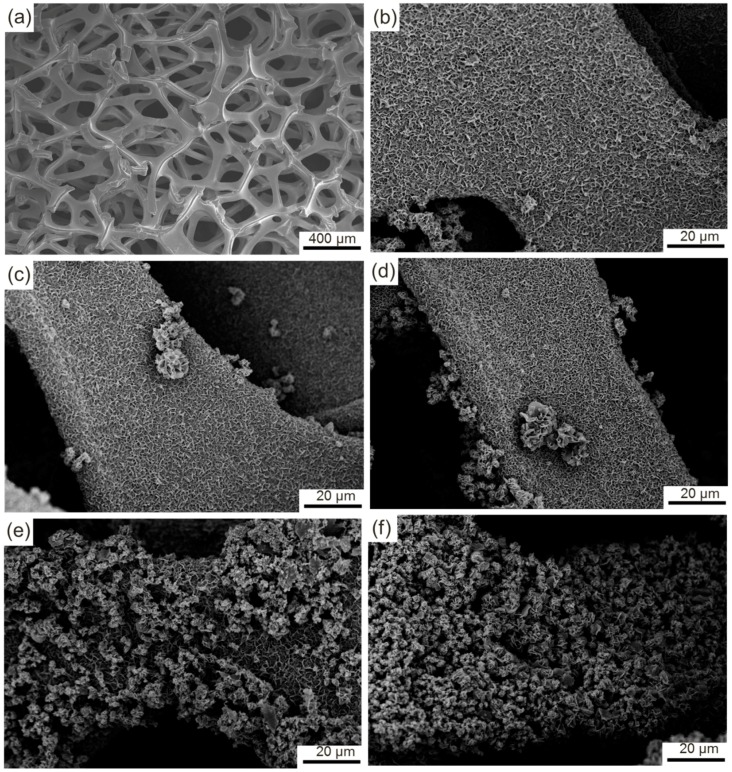
(**a**) SEM image of nickel foam; (**b–f**) Low magnification SEM images of samples B–F.

Scanning electron microscope (SEM) was used to observe the morphological evolution of the samples (Figure 9a–f). The nickel foam is composed of a large number of three-dimensional skeletons (Figure 9a), among which many holes can be clearly observed. After the hydrothermal reaction, the initial surface is completely covered with a thin layer of honeycomb-like products with loose and porous structures (Figure 9b). A small quantity of flower-like particles with large size protrude from the surfaces of the honeycomb-like products, and they are situated around the edges of the nickel foam and mainly result from the excessive growth of the products around these zones. With the further increases in HMT concentration to 6 and 8 mmol, the flower-like particles corresponding increase in size and quantity (Figure 9c,d). Most of the surfaces of the honeycomb-like products remain uncovered. However, when the HMT concentration is enhanced to 10 mmol, the morphology of the product exhibits a considerable change (Figure 9e). A large number of flower-like particles continue to grow and spread from the edge to the middle of the nickel foam. As a result, over 70% of the surface of the nickel foam is covered by these particles. The flower-like particles are predominant in the product. The honeycomb-like particles are completely transformed into flower-like particles at a HMT concentration of 12 mmol (Figure 9f). Close inspection reveals that the honeycomb-like particles possess a higher specific surface area than the flower-like particles.

**Figure 10:**
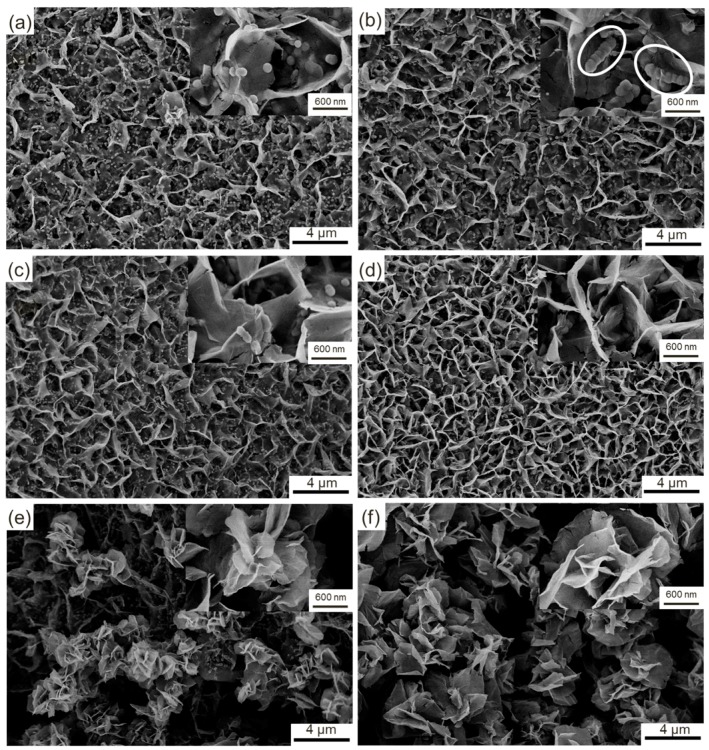
SEM images of the samples: (**a**) Sample A; (**b**) Sample B; (**c**) Sample C; (**d**) Sample D; (**e**) Sample E; (**f**) Sample F.

Additional morphological features are shown in high magnification SEM images (Figure 10a–f). Numerous nanosheets with a thickness of approximately 4 nm protrude from the comparatively flat surface and connect with one another, which results in the formation of the honeycomb-like structure (Figure 10a). Moreover, a large number of fine spheroidal particles with a diameter of approximately 100 nm are clearly observed. A high-magnification image, which is located in the upper right corner of Figure 10a, clearly indicates that these particles result from the excessive growth of partial zones. When the concentration of HMT is enhanced to 4 mmol, the nanoparticles grow to approximately 200 nm and cluster together (Figure 10b). As a result, some strips of protrusions similar to nanosheets are formed (white circle marked in Figure 10b). These protrusions grow into nanosheets with the increase in the concentration of HMT (Figure 10c). The independently dispersed nanoparticles nearly disappear and are completely transformed into nanosheets at a HMT concentration of 0.8 mmol (Figure 10d). The product surface is divided into small scattered zones with numerous nanosheets, among which a few narrow gaps are maintained. The evolution contributes to the increase in the specific area of the product. However, with the further increase in the concentration of HMT, the nanosheets continue to grow and are interconnected to form new growth sites, which finally grow into coarse flower-like particles (Figure 10e,f). Initial honeycomb-like particles are vaguely visible and completely covered with coarse flower-like particles at HMT concentrations of 1.0 and 1.2 mmol.

The morphological evolution can be intuitively described as follows. The fine nuclei that adhere to the surface of nickel foam constantly grow omnidirectionally and are gradually connected with one another, which results in the formation of a comparatively flat surface. As the reaction time is prolonged, the grain boundary with high energy grows preferentially compared with the grain. As a result, numerous fine nanosheets grow along the grain boundaries, thereby resulting in the formation of honeycomb-like structures. Nanosheets also present tiny changes in morphology due to the difference in concentration of reactants. The similar phenomenon also occurs at the flat zones surrounded by nanosheets. Several local zones with high growth rates preferentially protrude from the product surface and grow into fine nanoparticles. These spherical nanoparticles interconnect into long strip-like particles with HMT added into the solution along with initial nanosheets that grow constantly. A high concentration of HMT will hasten the growth of the long strip-like particles, which will result in the formation of sheet-like particles that are similar to the initial nanosheets. With the increase in density of nanosheets, new growth sites inevitably form and grow into flower-like particles. The specific area surface increases first and then decreases with the increase in HMT concentration. The highest specific area surface should be obtained from the product prepared with HMT/Co(NO_3_)_2_ at a mole ratio of 4:1.

The morphological evolution of the product is closely related to the concentration of HMT, which becomes a crucial affecting factor of the growth of the product. Obviously, a high HMT concentration offers a strong driving force to accelerate the growth of the nuclei. According to the synthesis of Co(OH)_2_ as described in Equations (1)–(4), HMT can sufficiently react with Co(NO_3_)_2_ to form Co(OH)_2_ at a mole ratio of 2:1. However, the opposite is true. HMT may be completely consumed. However, a portion of Co(NO_3_)_2_ may remain, which should be attributed to the important intermediate product (NH_3_). NH_3_ originates from the reaction between HMT and H_2_O, which further reacts with [Co(H_2_O)_6_]^2+^ to form [Co(NH_3_)_6_]^2+^. Finally, Co(OH)_2_ is synthesized by the reaction between [Co(NH_3_)_6_]^2+^ and OH^−^. A portion of NH_3_ escapes from the solution due to the highly volatile characteristic of NH_3_. Thus, the rest can only consume a portion of the added Co(NO_3_)_2_. That is, Co(NO_3_)_2_ may be excessive when HMT and Co(NO_3_)_2_ at a theoretical mole ratio of 2:1 is added into the solvent. Therefore, a higher mole ratio of HMT and Co(NO_3_)_2_ is very essential to ensure complete reactions between the two reactants. The evolution in morphology of the products also confirms that large amounts of Co(OH)_2_ are synthesized with the increase in the mole ratio from 1:1 to 6:1.

### 3.4. Electrochemical Characterization

**Figure 11:**
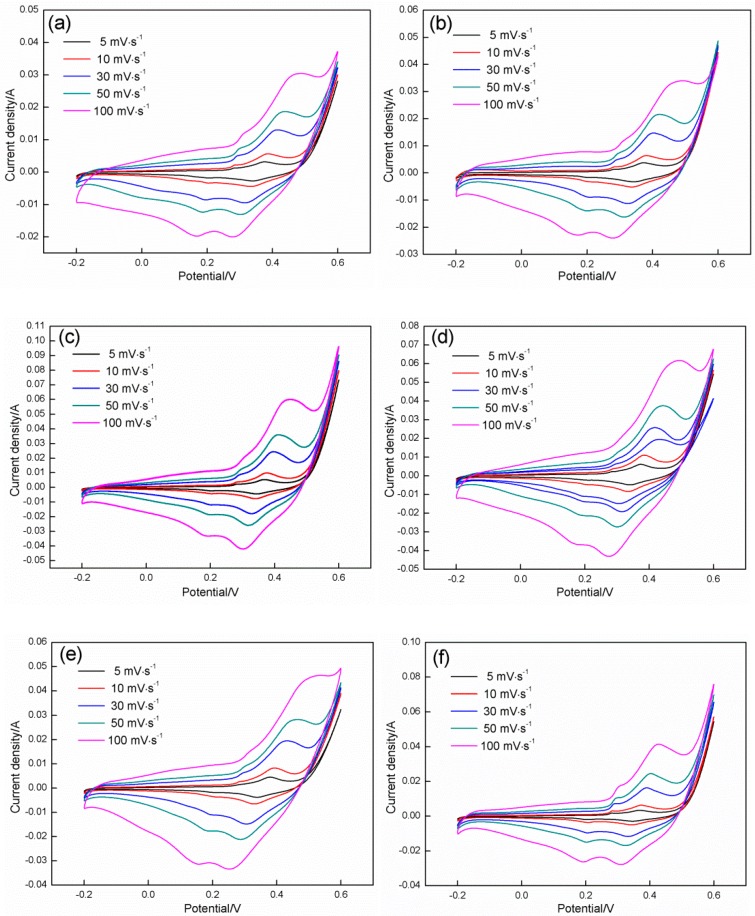
CV curves recorded on the samples at different scanning rates: (**a**) Sample A; (**b**) Sample B; (**c**) Sample C; (**d**) Sample D; (**e**) Sample E; (**f**) Sample F.

Figure 11 shows the CV results of the Co_3_O_4_ electrodes that were prepared on nickel foam at various HMT/Co(NO_3_)_2_ mole ratios. The scanning potential is swept from −0.2 V to 0.8 V, and the scanning rate is changed from 5 mV·s^−1^ to 100 mV·s^−1^. Notably, all obtained CV curves seriously deviate from the standard rectangle, which implies that the electrodes exhibit the typical pseudocapacitance behavior. The capacitance mainly originates from the redox reactions that occur between the active substance and electrolyte, which can be confirmed by the two clear redox peaks indicated in Figure 11. At a scanning rate of 100 mV·s^−1^, two oxidation peaks occur at 0.3 and 0.45 V along with two matching reduction peaks observed at 0.15 and 0.28 V, respectively. With the decrease in scanning rate from 100 mV·s^−1^ to 5 mV·s^−1^, the positions of the two oxidation peaks move to lower potentials, and the matching reduction peaks shift to higher potentials. The phenomenon may be attributed to the electrode reaction that seriously deviates from the equilibrium state with the increase in the scanning rate. The two redox reactions that occur on the electrode surface can be described as follows [45]:

(7) $${\mathrm{Co}}_{3}O_{4}+{\mathrm{OH}}^{-}+H_{2}O↔3\mathrm{CoOOH}+e^{-}$$

(8) $$\mathrm{CoOOH}+{\mathrm{OH}}^{-}↔{\mathrm{CoO}}_{2}+H_{2}O+e^{-}$$

The integrated area that is surrounded by the CV curves is closely related to the HMT/Co(NO_3_)_2_ mole ratio. With the increase in mole ratio of HMT/Co(NO_3_)_2_ from 1:1 to 4:1 at any given scanning rate, the area presents an increasing tendency. However, further increases in the mole ratio cause a reduction in the area. The area peak is acquired at a HMT/Co(NO_3_)_2_ mole ratio of 4:1. We can conclude that the capacitance can reach a maximum at a mole ratio of 4:1 due to the electrode capacitance that is proportionate to the integrated area. The calculated results of the specific capacitance further confirm this notion. The specific capacitances of Co_3_O_4_ can be calculated by integrating the area of the CV curves as follows [46]:

(9) $$C_{m}=\frac{1}{mv\left(∆V\right)}\int_{V_{a}}^{V_{c}}I\left(V\right)dV$$

where ∆V signifies the specific capacitance (F·g^−1^) as measured from the CV tests; *m* is the mass of Co_3_O_4_ that adheres to the nickel foam (g); ∆V denotes the potential range; and *V* and I(V) represent the scan rate (V·s^−1^) and corresponding current response, respectively.

Using the equation above, the specific capacitances of the six samples at various scanning rates are calculated. Sample D with a HMT/Co(NO_3_)_2_ mole ratio of 4:1 possesses the highest value (693.56 F·g^−1^) at a scanning rate of 5 mV·s^−1^, followed by Sample C with a mole ratio of 3:1 (559.86 F·g^−1^), Sample E with a mole ratio of 5:1 (423.35 F·g^−1^), Sample F with a mole ratio of 6:1 (321.64 F·g^−1^), Sample B with a mole ratio of 2:1 (217.33 F·g^−1^), and Sample A with a mole ratio of 1:1 (121.32 F·g^−1^). The same tendency can be observed using other scanning rates.

**Figure 12:**
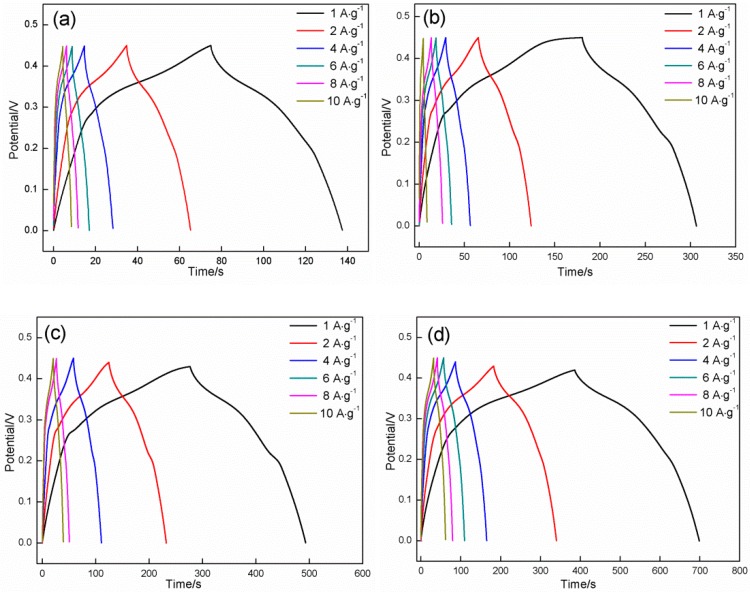

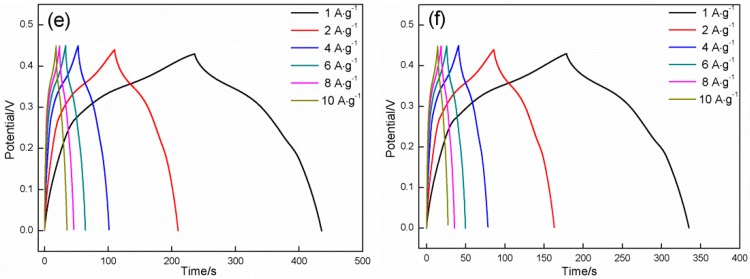
Galvanostatic charge/discharge curves recorded on the samples at different current densities: (**a**) Sample A; (**b**) Sample B; (**c**) Sample C; (**d**) Sample D; (**e**) Sample E; (**f**) Sample F.

Figure 12 presents the galvanostatic charge/discharge profiles of the products synthesized in different mole ratios at a potential range of 0–0.45 V with different charging and discharging current densities. These profiles are also deviated from a typical symmetrical triangular shape, and two charge/discharge platforms can be clearly observed (especially at a low current density). This result indicates that the products present a pseudocapacitance characteristic. The charge/discharge time is heavily reliant on the mole ratio of HMT/Co(NO_3_)_2_; specifically, it increases first and then decreases with the increase in mole ratio. The longest time period is obtained at a HMT/Co(NO_3_)_2_ mole ratio of 4:1, which indicates that the maximum specific capacitance is acquired at 4:1. This result agrees well with that confirmed in the CV tests. The specific capacitance of the products can be calculated using the following equation [47]:

(10) $$C_{m}=\frac{I\cdot ∆t}{m\cdot ∆v}$$

where $C_{m}$ is the specific capacitance measured from galvanostatic charge/discharge curve (F·g^−1^), *I* represents the constant discharge current (A), *m* is the mass of Co_3_O_4_ (g), ∆t (s) is discharge time and ∆v is the potential range (V).

The highest specific capacitances are also obtained in the product prepared at a HMT/Co(NO_3_)_2_ mole ratio of 4:1 at any given specific current density (743.00, 730.74, 710.93, 697.32, 680.88, and 621.21 F·g^−1^ at current densities of 1, 2, 4, 6, 8, and 10 A·g^−1^, respectively).

**Figure 13:**
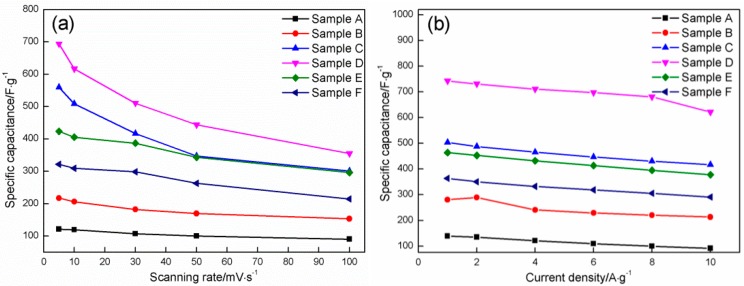
(**a**) Specific capacitance of six samples as a function of scanning rate; (**b**) Specific capacitance of six samples as a function of current density.

Figure 13 shows the specific capacitance of the six samples as a function of the scanning rate and current density. The specific capacitance decreases with the increases in the scanning rate and current density. The six samples retain approximately 74.44%, 70.55%, 53.77%, 51.16%, 70.66%, and 66.74% of the value obtained in 0.005 V·s^−1^ as the scan rate increases from 0.005 V·s^−1^ to 0.1 V·s^−1^ and approximately 65.93%, 76.06%, 82.87%, 83.61%, 81.46%, and 80.03% of the values obtained in 1 A·g^−1^ when the current density is increased from 1 A·g^−1^ to 10 A·g^−1^. These samples also present an outstanding rate performance. Generally speaking, sample D still possesses the highest specific capacitance when the scanning rate and the current density are improved from 0.005 to 0.1 V·s^−1^, and from 1 to 10 A·g^−1^, respectively.

The change in specific capacitance with HMT/Co(NO_3_)_2_ mole ratio is undoubtedly related to the above-mentioned morphological evolution, and it directly determines the specific surface area. A high specific surface area can provide added active sites for electron exchange and shorten the transfer path of the electrons and active ions. At a HMT/Co(NO_3_)_2_ mole ratio of 1:1, three zones with different morphologies coexist in the deposit surface and correspond to nanosheets, flat zones surrounded by nanosheets, and spherical nanoparticles that are uniformly distributed on the two zones, respectively. When the mole ratio of HMT/Co(NO_3_)_2_ increases to 2:1, striped particles are formed due to the growth and interconnection among spherical nanoparticles, which possess a lower specific surface area than that of the independently dispersed nanoparticles. However, the constant growth of nanosheets causes the increase in specific surface area. The latter plays a leading role in the change in specific surface area, thereby resulting in the increase in specific capacitance of the product (from 139.11 F·g^−1^ to 280.46 F·g^−1^ at 1 A·g^−1^, from 135.11 F·g^−1^ to 289.11 F·g^−1^ at 2 A·g^−1^, from 120.89 F·g^−1^ to 240.89 F·g^−1^ at 4 A·g^−1^, from 109.33 F·g^−1^ to 229.33 F·g^−1^ at 6 A·g^−1^, from 99.56 F·g^−1^ to 220.45 F·g^−1^ at 8 A·g^−1^, and from 91.72 F·g^−1^ to 213.33 F·g^−1^ at 10 A·g^−1^). The striped particles gradually grow into fine sheets along with the constant growth of initial nanosheets at a HMT/Co(NO_3_)_2_ mole ratio of 3:1, both of which will considerably enhance the specific surface area of the product. As a result, the specific capacitance of the product that is synthesized in 3:1 is increased to approximately 70–95% compared with that synthesized at 2:1. Tiny amounts of coarse flower-like particles with low specific surface areas are formed due to the connection and growth of nanosheets, and the effect of these particles on the specific surface area of the product is negligible. When the ratio is increased to 4:1, the flower-like particles produce an increasing effect on the specific area of the product due to their increase in number. However, the constant growth of nanosheets causes corresponding increases in specific surface area, which completely eliminates the reverse effect that result from the flower-like particles. As a consequence, specific capacitance is increased to 4:1. When the mole ratio is further enhanced to 8:1, the growth of the flower-like particles is predominant and nearly completely covers the entire surface of the product, which shields the initial nanosheets from fully coming into contact with the active ions. The specific capacitance is correspondingly reduced.

The change in rate performance of Co_3_O_4_ is closely related to the evolution in specific surface area of Co_3_O_4_ prepared in different mole ratios of HMT and Co(NO_3_)_2_. Co_3_O_4_ with a high specific surface area will provide more tunnels for the transportation of active ions and more active sites for the charge exchange, which can make a fast response to the change in charge-discharge rate due to the enhancement in charge-transfer rate. Moreover, the increase in specific surface area of Co_3_O_4_ will reduce its internal resistance due to the shortening in the electronic transfer path, which can also greatly enhance the charge-transfer rate when the charge-discharge rate is changed. Many investigations had confirmed that the increase in specific surface area contributed to the reduction in internal resistance. Zhang et al. [3] synthesized Co_3_O_4_ nanowire superstructure with different morphologies by a typical hydrothermal process. Three surfactants (polyvinyl pyrrolidone (PVP), cetyltrimethylammonium bromide (CTAB) and sodium dodecyl sulfate (SDS)) were applied to control its morphology. For comparison, a Co_3_O_4_ sample was synthesized under the same conditions but without addition of the surfactant. The EIS (electrochemical impedance spectroscopy) results indicated that the specific surface area of Co_3_O_4_ was closely related to its internal resistance. The SDS-Co_3_O_4_ sample with the highest specific surface area of 121.4 m^2^·g^−1^ possessed the lowest internal resistance of about 0.3 Ω, followed by the CTAB-Co_3_O_4_ sample (92.9 m^2^·g^−1^), the Co_3_O_4_ sample (85.6 m^2^·g^−1^) and the PVP-Co_3_O_4_ sample (65.0 m^2^·g^−1^) from low to high in internal resistance. Chen et al. [48] synthesized the nanostructured PbO_2_ thin films using a galvanostatic oxidation method to dilute the H_2_SO_4_ solution and investigated the effect of change in the morphology of PbO_2_ prepared at different current densities on electrochemical properties. The results also indicated that the sample with a high specific surface area exhibited a low internal resistance and excellent electrochemical properties. The other studies also confirmed this conclusion [28,49,50]. That is to say, a high specific surface area will endow the sample with a low internal resistance, which further causes the improvement in electrochemical property. In this study, the increase in tunnel/active site and the reduction in internal resistance resulting from the increase in specific surface area of Co_3_O_4_ are responsible for the improvement in its rate performance.

**Figure 14:**
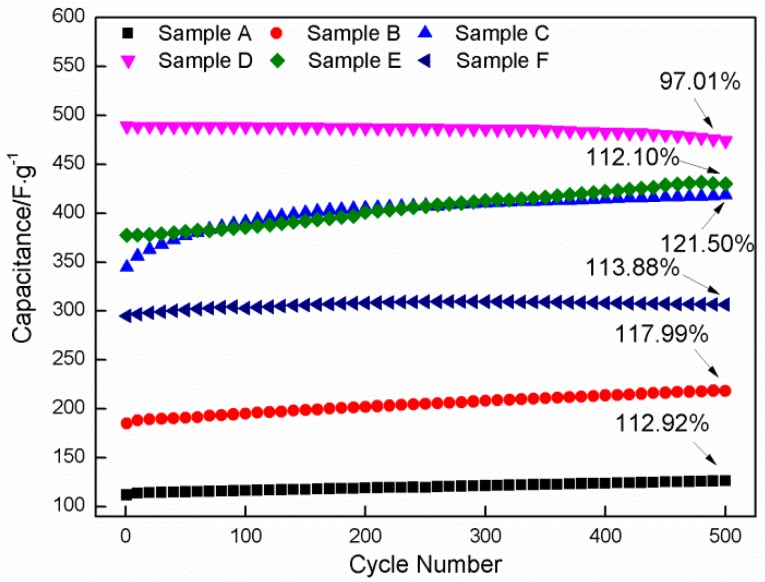
Cycling tests for Samples A–F at a scanning rate of 50 mV·s^−1^ up to 500 cycles.

The cycling stability of Co_3_O_4_ electrodes is evaluated by the CV tests for 500 cycles at 50 mV·s^−1^ (Figure 14). Clearly, the specific capacitance prepared at a HMT/Co(NO_3_)_2_ mole ratio of 4:1 is higher than those in other mole ratios. The specific capacitance of the sample prepared in 4:1 is 474.18 F·g^−1^ after 500 cycles, which indicates a capacitance retention of 97% from the initial value (488.77 F·g^−1^). Only a slight degradation (approximately 3%) is observed, which indicates that Sample D exhibits excellent cycling stability. However, the specific capacitances of the other samples are notably slightly increased after 500 cycling; their specific capacitance retentions are approximately 112.92% (Sample A), 117.99% (Sample B), 121.50% (Sample C), 112.10% (Sample E), and 113.88% (Sample F). The phenomenon is associated with the increase in active sites that participate in the electron transfer and the change in morphology resulting from the current shock and mechanical destruction during charge/discharge. An increased number of active sites will be activated as the electrolyte gradually infiltrates the electrode material during charge/discharge, which improves wettability and activation of the electrode. In addition, the active substance will be inevitably subject to repeated current shocks and mechanical destruction during cycling, from which the morphology of the active substance will be changed locally. For Samples A and B, the activation of the active sites is predominant in the increase in specific capacitance. Nanosheets with comparatively low height will tightly adhere to the electrode surface, which cannot be easily destroyed under the current shock due to the insufficient growth of the nanosheets prepared at low HMT/Co(NO_3_)_2_ mole ratios (1:1, 2:1). Therefore, the effect of the current shock on the specific capacitance of Samples A and B is negligible. Nanosheets with a large height protrude into the electrolyte when the HMT/Co(NO_3_)_2_ mole ratio is increased to 3:1, and they may be prone to breakage under the current shock. Accompanied by the fracture of the local nanosheets, the specific capacitance will be reduced. However, the activation of added active sites will cause an increase in specific capacitance. A slight increase in specific capacitance of Sample C, compared with that of Sample B, is a result of the integration of the two above-mentioned factors. With the further increase in mole ratio to 4:1, a strong current shock may be generated in Sample D with a considerably high specific surface area due to the frequent and violent electron transfer during charge/discharge. This phenomenon may accelerate the exfoliation of the nanosheets from the electrode surface during cycling. Current shock becomes an essential factor that causes the decrease in specific capacitance of the sample compared with the activation of active sites. With the increase in thickness of the deposits, the structural integrity cannot be well preserved after long time cycling due to the transformation reactions between Ni(OH)_2_ and NiOOH occurring on nickel foam [50,51]. The transformation reactions between Ni(OH)_2_ and NiOOH can cause the volumetric change of the surface layer of nickel foam, which will result in some Co_3_O_4_ particle detached from some local zones. For Sample D, since the new exposed area resulting from the destruction cannot compensate for the loss in area, the specific capacitance will be reduced to a certain extent. As a result, a slight reduction in specific capacitance occurs. When the HMT/Co(NO_3_)_2_ mole ratios are enhanced to 6:1 and 8:1, coarse flower-like particles that cover the nanosheets may fall off under the current shock, and nanosheets with a high specific surface area are exposed to the electrolyte, along with which the specific capacitance presents a tendency to increase with the increase in cycling number. For Samples E and F, the mechanical destruction will be greatly weakened, since it is very difficult for the electrolyte to permeate into nickel foam covered with the more compact deposits.

## 4. Conclusions

In summary, Co_3_O_4_ nanosheets with a high specific surface area were prepared on nickel foam by hydrothermal synthesis method followed by annealing treatment (300 °C). The mole ratio of HMT and Co(NO_3_)_2_·6H_2_O as the reactants was an essential factor determining the morphologies of Co_3_O_4_. The highest specific surface of Co_3_O_4_ prepared was obtained at a HMT and Co(NO_3_)_2_ mole ratio of 4:1, when their mole ratio was increased from 1:1 to 6:1. The specific capacitance of the electrode was closely related to the morphological evolution of Co_3_O_4_. The highest specific capacitance of the electrode was acquired for the product synthesized at a mole ratio of HMT and Co(NO_3_)_2_ (4:1) (743.00 F·g^−1^ at 1 A·g^−1^ in galvanostatic charge/discharge tests, 693.56 F·g^−1^ at 5 mV·s^−1^ in CV tests). Co_3_O_4_ synthesized in the mole ratio of HMT and Co(NO_3_)_2_ (4:1) exhibited excellent cyclic performance; about 97% of the initial specific capacitance remained after 500 cycling tests.

## Figures and Tables

**Table 1:** The specific capacitance of Co_3_O_4_ reported in some related references.

Materials	Specific Capacitance	Cycle Performance	References
Co_3_O_4_ microspheres	850.00 F·g^−1^/1 A·g^−1^	90.8%/1000 cycles/2 A·g^−1^	[12]
Co_3_O_4_ microspheres	261.10 F·g^−1^/0.5 A·g^−1^	90.2%/2000 cycles/5 A·g^−1^	[27]
Co_3_O_4_ octahedras	98.00 F·g^−1^/1 A·g^−1^	195.9%/3000 cycles/2 A·g^−1^	[28]
Co_3_O_4_ particulates	224.38 F·g^−1^/2.75 A·g^−1^	72.2%/1000 cycles/2.75 A·g^−1^	[29]
Co_3_O_4_ nanoparticles	157.00 F·g^−1^/0.5 A·g^−1^	100%/2500 cycles/1 A·g^−1^	[30]
Co_3_O_4_ nanowires	599.00 F·g^−1^/20 A·g^−1^	91%/7500 cycles/2 A·g^−1^	[13]
Co_3_O_4_ nanowires	439.00 F·g^−1^/40 A·g^−1^	82%/7500 cycles/10 A·g^−1^	[13]
Co_3_O_4_ nanowires	471.80 F·g^−1^/0.5 A·g^−1^	94.8%/1000 cycles/2 A·g^−1^	[14]
Co_3_O_4_ nanorods	655.00 F·g^−1^/0.5 A·g^−1^	82.7%/1000 cycles/3 A·g^−1^	[15]
Co_3_O_4_ nanorods	352.00 F·g^−1^/1 A·g^−1^	129.6%/500 cycles/1 A·g^−1^	[31]
Co_3_O_4_ flakes	263.00 F·g^−1^/20 A·g^−1^	89.4%/1000 cycles/3 A·g^−1^	[17]
Co_3_O_4_ flakes	450.00 F·g^−1^/1 A·g^−1^	92%/5000 cycles/1 A·g^−1^	[32]
Co_3_O_4_ films	325.00 F·g^−1^/2 A·g^−1^	139.69%/2500 cycles/2 A·g^−1^	[16]
Co_3_O_4_ nanoplates	231.00 F·g^−1^/1 A·g^−1^	97%/2000 cycles/2 A·g^−1^	[33]

## References

[1] Li G., Liu W.L., Wang Z.H., Liu M.Q. (2017). An empirical examination of energy consumption, behavioral intention, and situational factors: Evidence from Beijing. Ann. Oper. Res..

[2] Parvini Y., Siegel J.B., Stefanopoulou A.G., Vahidi A. (2016). Supercapacitor electrical and thermal modeling, identification, and validation for a wide range of temperature and power applications. IEEE Trans. Ind. Electron..

[3] Zhang X., Zhao Y.Q., Xu C.L. (2014). Surfactant dependent self-organization of Co_3_O_4_ nanowires on Ni foam for high performance supercapacitors: From nanowire microspheres to nanowire paddy fields. Nanoscale.

[4] Huang W.X., Li J., Xu Y.H. (2017). Nucleation/Growth mechanisms and morphological evolution of porous MnO_2_ coating deposited on graphite for supercapacitor. Materials.

[5] Vezvaie M., Kalisvaart P., Fritzsche H., Tun Z., Mitlin D. (2014). The penetration depth of chemical reactions in a thin-film Co_3_O_4_ supercapacitor electrode. J. Electrochem. Soc..

[6] Wee G., Soh H.Z., Cheah Y.L., Mhaisalkar S.G., Srinivasan M. (2010). Synthesis and electrochemical properties of electrospun V_2_O_5_ nanofibers as supercapacitor electrodes. J. Mater. Chem..

[7] Meng X.Q., Zhou M., Li X.L., Yao J.Y., Liu F.L., He H.C., Xiao P., Zhang Y.H. (2013). Synthesis of SnO_2_, nanoflowers and electrochemical properties of Ni/SnO_2_, nanoflowers in supercapacitor. Electrochim. Acta.

[8] Ambade R.B., Ambade S.B., Shrestha N.K., Nah Y.C., Han S.H., Lee W., Lee S.H. (2013). Polythiophene infiltrated TiO nanotubes as high-performance supercapacitor electrodes. Chem. Commun..

[9] Patil U.M., Salunkhe R.R., Gurav K.V., Lokhande C.D. (2008). Chemically deposited nanocrystalline NiO thin films for supercapacitor application. Appl. Surf. Sci..

[10] Meng T., Xu Q.Q., Wang Z.H., Li Y.T., Gao Z.M., Xing X.Y., Ren T.Z. (2015). Co_3_O_4_ nanorods with self-assembled nanoparticles in queue for supercapacitor. Electrochim. Acta.

[11] Li T.T., Zhu C.X., Yang X.G., Gao Y.H., He W.W., Yue H.W., Zhao H.X. (2017). Co_3_O_4_ nanoneedle@electroactive nickel boride membrane core/shell arrays: A novel hybrid for enhanced capacity. Electrochim. Acta.

[12] Feng C., Zhang J.F., Deng Y.D., Zhong C., Liu L., Hu W.B. (2015). One-pot fabrication of Co_3_O_4_, microspheres via hydrothermal method at low temperature for high capacity supercapacitor. Mater. Sci. Eng. B.

[13] Xia X.H., Tu J.P., Mai Y.J., Wang X.L., Gu C.D., Zhao X.B. (2011). Self-supported hydrothermal synthesized hollow Co_3_O_4_ nanowire arrays with high supercapacitor capacitance. J. Mater. Chem..

[14] Chang B.B., Gu Z.Y., Guo Y.Z., Li Z.K., Yang B.C. (2016). Glucose-assisted synthesis of Co_3_O_4_, nanostructure with controllable morphologies from nanosheets to nanowires. J. Alloy. Compd..

[15] Venkatachalam V., Alsalme A., Alswieleh A., Jayavel R. (2018). Shape controlled synthesis of rod-like Co_3_O_4_, nanostructures as high-performance electrodes for supercapacitor applications. J. Mater. Sci. Mater. Electron..

[16] Liu W., Jiang D., Xia J.X., Qian J., Wang K., Li H.M. (2014). Preparation of hierarchical mesoporous Co_3_O_4_, bundle using [Bmim]TA as a multi-role starting material and its supercapacitor application. Monatsh. Chem..

[17] Xie L.J., Li K.X., Sun G.H., Hu Z.G., Lv C.X., Wang J.L., Zhang C.M. (2013). Preparation and electrochemical performance of the layered cobalt oxide (Co_3_O_4_) as supercapacitor electrode material. J. Solid State Electrochem..

[18] Duan B.R., Cao Q. (2012). Hierarchically porous Co_3_O_4_ film prepared by hydrothermal synthesis method based on colloidal crystal template for supercapacitor application. Electrochim. Acta.

[19] Zhao J.W., Zheng Z.L., He X.W., Geng W.C. (2017). Advances in hydrothermal synthesis of Co_3_O_4_ nanomaterials in different dimensions. Bull. Chem..

[20] Farhadi S., Javanmard M., Nadri G. (2016). Characterization of cobalt oxide nanoparticles prepared by the thermal decomposition. Acta Chim. Slov..

[21] Yang Y.P., Huang K.L., Liu R.S., Wang L.P., Zeng W.W., Zhang P.M. (2007). Shape-controlled synthesis of nanocubic Co_3_O_4_ by hydrothermal oxidation method. Trans. Nonferr. Met. Soc. China.

[22] Büyükyazi M., Hegemann C., Lehnen T., Tyrra W., Wathur S. (2014). Molecular Co (II) and Co (III) heteroarylalkenolates as efficient precursors for chemical vapor deposition of Co_3_O_4_ nanowires. Inorg. Chem..

[23] Hwa Y., Kim W.S., Yu B.C., Hong S.H., Sohn H.J. (2013). Enhancement of the cyclability of a Si anode through Co_3_O_4_ coating by the Sol–Gel method. J. Phys. Chem. C.

[24] Jagadale A.D., Kumbhar V.S., Bulakhe R.N., Lokhande D.C. (2014). Influence of electrodeposition modes on the supercapacitive performance of Co_3_O_4_ electrodes. Energy.

[25] Feng Y.L., Liu Y.F., Su C., Ji X.H., He Z.K. (2014). New fluorescent pH sensor based on label-free silicon nanodots. Sens. Actuators B Chem..

[26] Du W., Liu R.M., Jiang Y.W., Liu Q.Y., Fan Y.Z., Gao F. (2013). Facile synthesis of hollow Co_3_O_4_ boxes for high capacity supercapacitor. J. Power Sources.

[27] Guo D.X., Song X.M., Li F.F., Tan L.C., Ma H.Y., Zhang L.L., Zhao Y.Q. (2018). Oriented synthesis of Co_3_O_4_ core-shell microspheres for high-performance asymmetric supercapacitor. Colloid Surf. A.

[28] Cao Y.B., Yuan F.L., Yao M.S., Bang J.H., Lee J.H. (2013). A new synthetic route to hollow Co_3_O_4_ octahedra for supercapacitor applications. CrystEngComm.

[29] Tummala R., Guduru R.K., Mohanty P.S. (2012). Nanostructured Co_3_O_4_ electrodes for supercapacitor applications from plasma spray technique. J. Power Sources.

[30] Xu W., Li T.T., Zheng Y.Q. (2016). Porous Co_3_O_4_ nanoparticles derived from Co (II)-cyclohexanehexacarboxylate metal-organic framework used as supercapacitor with good cycling stability. RSC Adv..

[31] Li C., Li J., Zhang X.G. (2009). Preparation and properties of Co_3_O_4_, nanorods as supercapacitor material. J. Appl. Electrochem..

[32] Xiao A.G., Zhou S.B., Zuo C.G., Zhuan Y.B., Ding X. (2014). Controllable synthesis of mesoporous Co_3_O_4_ nanoflake array and its application for supercapacitor. Mater. Res. Bull..

[33] Geng T., Zhang L., Wang H.Y., Zhang K.Y., Zhou X. (2015). Facile synthesis of porous Co_3_O_4_ nanoplates for supercapacitor applications. Bull. Mater. Sci..

[34] Du H.M., Li Y.Y., Ding F.F., Zhao J.S., Zhang X.X., Li Y.W., Zhao R.J., Cao M.T., Yu T.T., Xu X.J. (2018). Boosting the capacitance of NiCo_2_O_4_ hierarchical structures on nickel foam in supercapacitors. Int. J. Hydrog. Energy.

[35] Cai D.P., Xiao S.H., Wang D.D., Liu B., Wang L.L., Liu Y., Li H., Wang Y.R., Li Q.H., Wang T.H. (2014). Morphology controlled synthesis of NiCo_2_O_4_ nanosheet array nanostructures on nickel foam and their application for pseudocapacitors. Electrochim. Acta.

[36] Wang X.H., Shi B., Huang F.F., Fang Y., Rong F., Que R.H. (2018). Fabrication of hierarchical NiCo_2_O_4_@NiCo_2_S_4_ core/shell nanowire arrays by an ion-exchange route and application to asymmetric supercapacitors. J. Alloy. Compd..

[37] Peiris T.A.N., Senthilarasu S., Wijayantha K.G.U. (2012). Enhanced performance of flexible dye-sensitized solar cells: Electrodeposition of Mg(OH)_2_ on a nanocrystalline TiO_2_ electrode. J. Phys. Chem. C.

[38] Grujicic D., Pesic B. (2004). Electrochemical and AFM study of cobalt nucleation mechanisms on glassy carbon from ammonium sulfate solutions. Electrochim. Acta.

[39] Lane G.H., Jezek E. (2014). Electrochemical studies of acetonitrile based supercapacitor electrolytes containing alkali and alkaline earth metal cations. Electrochim. Acta.

[40] Sun W.Y. (2010). Coordination Chemistry.

[41] Feng C., Zhang J., He Y., Zhong C., Hu W., Liu L., Deng Y. (2015). Sub-3 nm Co_3_O_4_ nanofilms with enhanced supercapacitor properties. ACS Nano.

[42] Morgan K.R., Gainsford G.J., Milestone N.B. (1997). A new type of layered aluminium phosphate [NH_4_]_3_[Co(NH_3_)_6_]_3_[Al_2_(PO_4_)_4_]_2_ assembled about a cobalt(iii) hexammine complex. Chem. Commun..

[43] Hoshino K., Hitsuoka Y. (2005). One-step template-free electrosynthesis of cobalt nanowires from aqueous [Co(NH_3_)_6_]Cl_3_ solution. Electrochem. Commun..

[44] Ding K., Zhang X., Yang P., Cheng X. (2016). A precursor-derived morphology controlled synthesis method for mesoporous Co_3_O_4_ nanostructures towards supercapacitor application. CrystEngComm.

[45] Wang J.Y., Dou W., Zhang X.T., Han W.H., Mu X.M., Zhang Y., Zhao X.H., Chen Y.X., Yang Z.W., Su Q. (2017). Embedded Ag quantum dots into interconnected Co_3_O_4_ nanosheets grown on 3D graphene networks for high stable and flexible supercapacitors. Electrochim. Acta.

[46] Barmi A.A.M., Aghazadeh M., Arhami B., Shiri H.M., Fazl A.A., Jangju E. (2012). Porous cobalt hydroxide nanosheets with excellent supercapacitive behavior. Chem. Phys. Lett..

[47] Xu Y.H., Li J., Huang W.X. (2017). Porous Graphene Oxide Prepared on Nickel Foam by Electrophoretic Deposition and Thermal Reduction as High-Performance Supercapacitor Electrodes. Materials.

[48] Chen T., Huang H., Ma H.Y., Kong D.L. (2013). Effects of surface morphology of nanostructured PbO_2_ thin films on their electrochemical properties. Electrochim. Acta.

[49] Gao L.B., Xu S., Xue C.Y., Hai Z.Y., Sun D., Lu Y. (2016). Self-assembly of hierarchical 3D starfish-like Co_3_O_4_ nanowire bundles on nickel foam for high-performance supercapacitor. J. Nanopart. Res..

[50] Fan H.Q., Quan L.X., Yuan M.Q., Zhu S.S., Wang K., Zhong Y., Chang L., Shao H.B., Wang J.M., Zhang J.Q. (2016). Thin Co_3_O_4_, nanosheet array on 3D porous graphene/nickel foam as a binder-free electrode for high-performance supercapacitors. Electrochim. Acta.

[51] Kong D.S., Wang J.M., Shao H.B., Zhang J.Q., Cao C.N. (2011). Electrochemical fabrication of a porous nanostructured nickel hydroxide film electrode with superior pseudocapacitive performance. J. Alloys Compd..

